# Study planning is better served by Monte Carlo simulation than AI-generated participants

**DOI:** 10.1038/s44271-026-00556-y

**Published:** 2026-11-11

**Authors:** Jamie Cummins

**Affiliations:** 1 Institute of Psychology, University of Bern, Bern, Switzerland; 2 Bennett Institute for Applied Data Science, University of Oxford, Oxford, UK

**Keywords:** Human behaviour, Sociology, Economics

## Abstract

‘Silicon samples’, AI-generated synthetic data based on demographic profiles, have been proposed to help plan research. Here, I argue that Monte Carlo simulation already does this job transparently, and that generative AI is better used to help write such simulations.

Two researchers are planning the same experiment. Neither has begun data collection, and both want to adhere to best practices in study planning: specifying in advance the required sample size to achieve sufficient power, informed by a plausible effect size and expected spread of responses. The first writes a Monte Carlo simulation: for each combination of effect size, variance, and sample size, they generate 10,000 complete datasets and apply the planned analysis. They then estimate power for this analysis in each case and choose a sample size that offers at least 80% power across the scenarios they consider plausible. The second prompts a large language model (LLM) to respond to the scales of interest 10,000 times, providing a distinct demographic persona each time. They run their planned analysis on this LLM-generated data, treat the observed effect size as an estimate of the human effect, and use it to motivate a subsequent power analysis. They then adopt the sample size this analysis produces. Which approach provides the more defensible basis for planning?

This question has come to prominence in the social and behavioral sciences, as a growing body of work advocates that “silicon samples” could be useful in research planning^1,2^. A recent Nature paper demonstrated that LLM-derived predictions correlated strongly with treatment effects across 70 nationally representative survey experiments (although systematically overestimating their magnitudes), with pilot testing as one of the central applications explored^3^. An accompanying commentary drew the natural conclusion: such models could serve as low-cost tools to assist experimental design^4^. Even sceptics of silicon samples might grant that they could cheaply help with pretesting or planning of study designs. The question is what status this method has relative to Monte Carlo simulation when determining the design of a study.

## Study planning with Monte Carlo simulation

Monte Carlo simulation-based study planning^5^ is routine in the social and behavioral sciences. A researcher first specifies a data-generating process (DGP) they consider plausible for the phenomenon of interest. Relevant quantities might include the assumed effect size, an error term such as ${\varepsilon }_{i}\, \sim {N}(0,\,{\sigma }^{2})$, and dependencies among observations. The researcher then defines plausible levels of the DGP and design parameters and repeatedly simulates complete experiments under each condition. For power planning, the proportion of simulated experiments in which the intended statistical decision is reached estimates power. Comparing conditions identifies the designs that achieve acceptable power across the range of assumptions considered plausible.

The value of Monte Carlo simulation for study planning comes from two properties. First, claims from simulations rest on transparent assumptions. With Monte Carlo simulation we say: if the world is like this, the design will have these properties. Simulation may be guesswork, but it is principled and transparent guesswork. Second, simulations are not tied to exploring only one set of assumptions. If a researcher is unsure whether an effect will be a Cohen’s d of 0.5 or 0.1, or an intraclass correlation 0.05 or 0.20, they can simply explore these possibilities directly. Planning under ignorance is exactly the benefit of using Monte Carlo simulation.

## Study planning with silicon samples

Silicon samples in study planning have been proposed as a way of estimating the quantities that Monte Carlo simulation requires. They therefore approach the same planning problem from an inverted epistemological position. Rather than specify a DGP and ask what follows, the researcher treats the distribution induced by the LLM as evidence about the human DGP^6^. The researcher can manipulate the interface to this learned mechanism (e.g., the prompt formatting and demographic information provided) but can neither directly inspect it nor alter it.

Silicon samples not only assume the DGP, but also the parameters of relevance to it. More specifically, silicon sampling focuses on demographic features and personal information, thereby assuming that this provides sufficient signal to make predictions about some outcome (e.g., responses on a novel attitude scale). Theory or domain expertise can guide to some extent which demographic variables should be included, but no general method exists that determines how such information should best be presented in a prompt or how to ensure that the model uses it appropriately. The grammar of statistical modeling, by contrast, provides a much clearer guide: a Monte Carlo simulation includes an intraclass correlation because the statistics of clustered designs demand it; a silicon sample includes age or political orientation because the researcher suspects it may be relevant.

Silicon sampling similarly says little about how prompting itself should be structured. In a Monte Carlo simulation, semantically equivalent implementations of the same specified DGP target the same distribution; if they do not, there is a substantive difference in implementation that can be identified and fixed. In a silicon sample, the formal properties of the prompt actively influence the data-generating mechanism. Choices that are substantively irrelevant to the phenomenon of interest (e.g., formatting a persona as prose or as bullet points, or rephrasing an instruction) can shift model output substantially, and no principled approach specifies which prompt features matter, by how much, or how they should be fixed. Silicon samples therefore come with additional, nonspecific “nuisance parameters” which are unrelated to the phenomenon of interest and that the researcher cannot enumerate in advance.

**Fig. 1:**
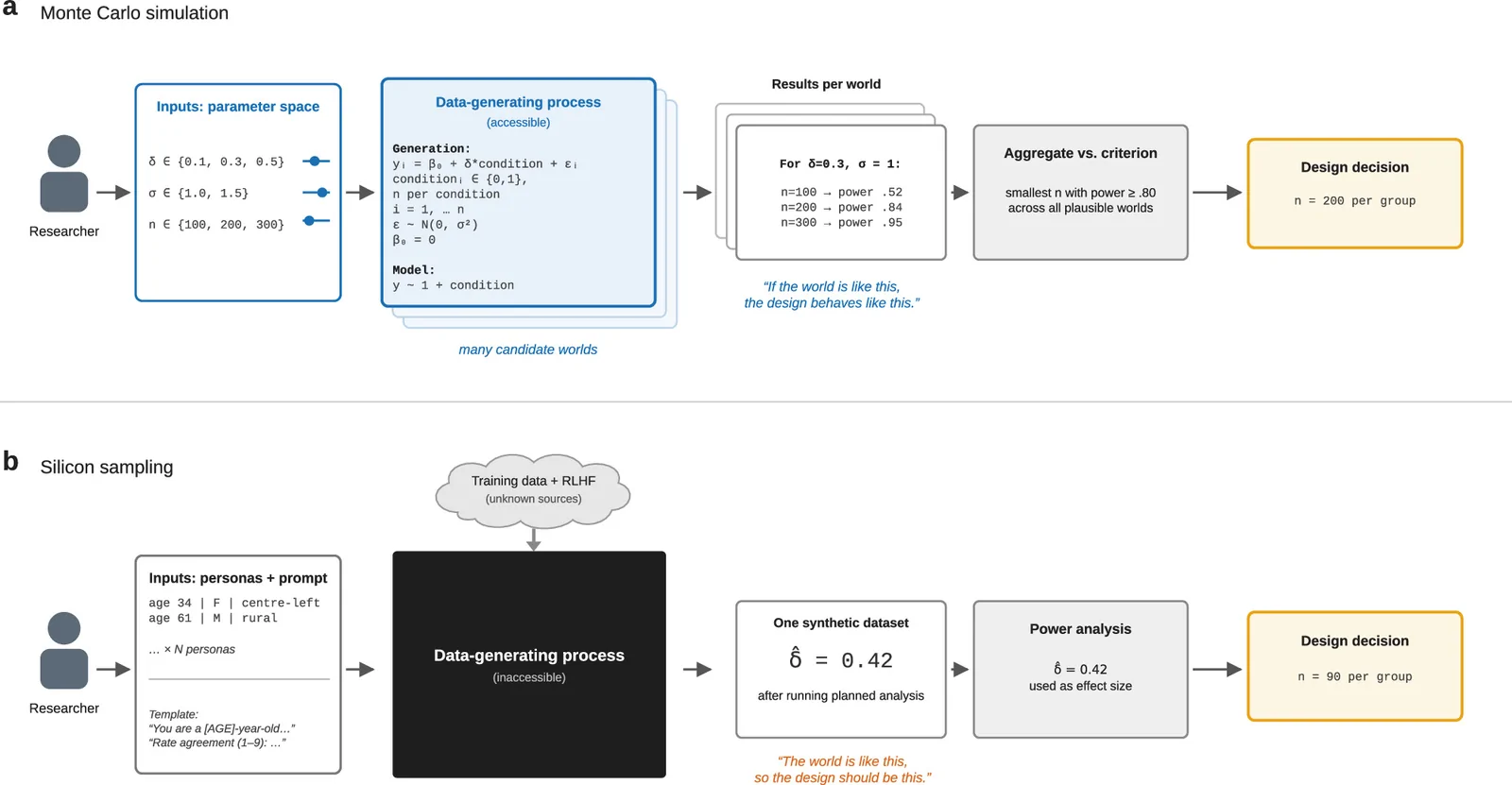
A contrast of study planning via Monte Carlo simulation and silicon sampling. **a** Monte Carlo simulation. The researcher specifies a parameter space of plausible values. Each combination of parameters defines one candidate world, which is provided to the fully accessible data-generating process specified in code and statistical notation. Repeated simulations across each of these plausible worlds are then aggregated to provide information about the behaviour of the design in each world (e.g., power). These results are used by the researcher (e.g., compared against a pre-specified criterion) to arrive at a specific design decision. **b** Silicon sampling. The researcher specifies inputs in the form of demographic personas and a prompt template to be used. The data-generating process is unknown, inaccessible, and is a function of the language model's training. Sampling from this process over personas produces a single synthetic dataset, and applying the planned analysis returns a statistical estimate (in this case, an effect size estimate). This estimate is then carried into a conventional power analysis as an assumed proxy for the human data, and the result from the power analysis then informs the researcher’s design decision.

Silicon sample-based study planning therefore carries an additional assumption beyond those made explicit in conventional Monte Carlo-based planning: that the model-induced distribution approximates the human one. For a fixed configuration *c*, let ${\theta }_{c}$ be the value a planning quantity (e.g., an effect size) would take under unlimited generation, and ${\theta }_{H}$ its value in the human population. A silicon sample estimate can miss the true human value by two gaps: finite silicon-sample error, which shrinks as more synthetic observations are generated, and model-human discrepancy, which does not. Under ordinary independent-sampling conditions, generating 100 times as many answers makes the first gap roughly 10 times smaller, whereas the second gap is unaffected. A Monte Carlo simulation-based planning approach says: if the world is like this, this would be the consequence. A silicon sample-based planning approach says: the world is like this, this is the consequence. The first claim is conditional and knows it; the second is conditional and forgets it (Fig. 1).

## Configuration dependence and generalizability in silicon samples

One might reasonably argue that opacity of the DGP is unimportant if silicon samples nevertheless predict human outcomes successfully: perhaps we already have access to the correct DGP without knowing its nature. This position holds only insofar as the silicon sample matches humans on the specific planning quantity of interest. Recent evidence suggests that this is frequently not the case: a model can recover one feature of human data while erring systematically on another^7,8^. Moreover, there is no single silicon sample DGP: the outputs of silicon samples vary drastically depending on the model selected, hyperparameter decisions (e.g., temperature or reasoning effort), and the content of prompts^7,9^. In this sense, each such configuration comes with its own distinct DGP, each of which is inaccessible and therefore cannot be directly compared in the same structural terms as two explicitly specified DGPs in a Monte Carlo simulation.

This is compounded by the fact that a silicon sample’s accuracy also does not necessarily generalize across assessment contexts: a configuration can recover a feature of human data well in one domain and poorly in another, so one “validated” for a previous study cannot be presumed adequate for the study being planned^7^. Consequently, two researchers using two different, equally defensible silicon sample configurations may obtain markedly different pilot estimates and make markedly different planning decisions. Because we cannot inspect the silicon sample DGP or its assumptions, the model outputs alone provide no way of determining why these decisions diverged or which DGP is more principled. By contrast, researchers who specify different Monte Carlo DGPs can inspect and debate their assumptions directly.

## Meaningfulness of variation

Good Monte Carlo simulations model not only the signal of interest but also relevant sources of variation, such as measurement error. If an error term is specified as ${\varepsilon }_{i}\, \sim {N}(0,\,{\sigma }^{2})$, its distribution and variance are explicit assumptions within the simulation. Varying ${\sigma }^{2}$ asks what would happen to the design if participant responses or measurements were noisier. Silicon samples contain both variation across personas and stochastic variation across generations from the same persona. The latter can be altered through the temperature hyperparameter, which changes the stochasticity of token sampling and thereby alters the variability of the LLM’s responses^10^. However, unlike varying ${\sigma }^{2}$, varying temperature does not set survey-response variance directly. Varying temperature asks what happens when an LLM’s decoder changes its sampling pattern, but this question is about the language model, not the planned study. This is especially consequential when a silicon sample-based pilot produces a standardized effect size. This estimated effect size can change because the model predicts a particular effect of interest, because the decoder produces different response dispersion, or both. The estimated effect is therefore a property of the model configuration and is not itself evidence about human variation in the DGP.

## Accumulation

Study planning is also where prior knowledge should accumulate. Monte Carlo simulation can incorporate effect estimates, measurement reliabilities, intraclass correlations and unpublished data explicitly, typically as fixed values, constrained ranges or distributions. Silicon sampling provides no comparably explicit mechanism. Researchers could in principle supply prior findings directly through prompts, but it is not possible to determine what weight the model gives this evidence or how it interacts with its existing training data.

## Language models as aids to Monte Carlo simulation

Readers should note that none of the above implies that LLMs have no role in simulation-based planning. For example, it is certainly conceivable that researchers may use agentic coding tools to assist in the development of code which executes a Monte Carlo simulation, thereby lowering the technical barriers which historically may have prevented researchers from conducting such simulations. Of course, this use is distinct from silicon sampling, since the LLM does not serve as the data-generating mechanism. Further, the benefits of simulation are preserved only if the researcher themselves inspects, justifies, and varies the assumptions which are embedded in that simulation. If an LLM also selects the parameters used in the simulation with no oversight from the researcher, then these model-selected values are in themselves of unknown practical relevance (analogous to a Bayesian analysis with unexamined priors). If using LLMs to generate Monte Carlo simulation code, this code must therefore be checked to ensure that it indeed implements the intended DGP and does so using parameters which are of practical relevance for the study being planned.

## Conclusion

Could LLMs provide utility during research planning and piloting? In their own words: Certainly! LLMs can help assess whether instructions are comprehensible, identify potentially ambiguous scale items, or otherwise suggest revisions to study materials. These uses are distinct from silicon sampling, which is the specific case of using LLMs to serve as proxies for human quantitative data. LLMs may well have uses in piloting. Silicon sampling for effect-size or sample-size planning should not be among them.

Let us return to the two researchers from the outset. Both used synthetic data, but the first can specify, justify and vary the assumptions on which their planning decisions depend. The second can document the model and prompt they used, but they cannot describe the DGP these features enact computationally. Researchers planning their studies will benefit from sticking to the tried-and-true method of Monte Carlo simulation: making assumptions about people transparently, rather than burying them in a model.

## Supplementary information

Transparent Peer Review file

## Data Availability

There are no primary research data associated with this publication.
