# Supplementary material for: Study planning is better served by Monte Carlo simulation than AI-generated participants
Source: Commun Psychol. 2026 Nov 11;4:160. doi: 10.1038/s44271-026-00556-y (PMC13754819; doi:10.1038/s44271-026-00556-y)
Supplement: Supplementary file 1 [file 44271_2026_556_MOESM1_ESM.pdf]

nature portfolio

# Peer Review File

# Study planning is better served by Monte Carlo simulation than AI-generated participants

Corresponding Author: Dr Jamie Cummins

**This file contains all reviewer reports in order by version, followed by all author rebuttals in order by version.**

Version 0:

Reviewer comments:

Reviewer #1

(Remarks to the Author)
The authors present a compelling argument for the transparency of MC simulations compared with the often opaque assumptions embedded in AI-generated "silicon samples." However, a central limitation of the current manuscript is its narrow definition of how Large Language Models (LLMs) function and are utilized by researchers.

I strongly recommend addressing the following points to nuance the paper's core argument:
The manuscript currently evaluates LLMs as if they were strictly in-context language generators, in which the model is prompted to directly output a synthetic dataset as text, relying solely on its internal weights and probabilistic next-token prediction. In this specific "naive" use case, the authors are entirely correct that the assumptions are buried, and the data-generation process is opaque. So the model did not generate or process any data, but just provided the most plausible anwer.

Or, did the model translate the prompt into python code and actually run a simulation in back-end? So, the output is based on an actual simulation study, only the parameters are not shown to the user. A simple prompt instruction will solve this problem:

I would suggest that the authors acknowledge that modern LLMs are frequently used to generate, execute, and document actual code. If a researcher prompts a current-generation model to create a synthetic demographic dataset, a standard workflow is for the model to translate this prompt into a Python script, run a Monte Carlo simulation in the backend, and output the results alongside a fully documented GitHub repository, complete with scripts, adjustable parameters, and a README. All of this is stored at your laptop and be pushed to GitHub.

Because an LLM can trivially generate a transparent, reproducible Monte Carlo pipeline, treating "Silicon Samples via LLM" and "Monte Carlo Simulation" as mutually exclusive categories is a false dichotomy. The issue is not that LLMs cannot provide transparency; the issue is how researchers choose to interact with them. Therefore, my recommendation is to that the authors should consider shifting the focus of their critique toward researcher methodology. The paper would be significantly stronger if it cautioned against the "naive" use of LLMs for direct, unstructured data generation, while acknowledging that LLMs are highly effective tools for generating the very transparent Monte Carlo simulation scripts the authors rightfully advocate for.

To strengthen the narrative, the authors might consider drawing a parallel between the current naive use of LLMs and the historical (and ongoing) misuse of p-values or Bayesian statistics. Just as a p-value is mathematically sound but frequently misused as a "black box" metric for truth (e.g., p-hacking) due to a lack of statistical literacy, LLMs are currently being misused as magic data generators. Similarly, Bayesian statistics require highly transparent justifications for priors. When researchers fail to document their priors, the Bayesian method becomes exactly what the authors fear LLMs are: an opaque container for unexamined assumptions.

Ultimately, I am afraid the core problem lies not with the tool, but, ingain with the human usr. Asen the ca,se with new sta,tistical tools, the danger lies not within the technology itself, but rather in how we as researchers choose to apply it.

**Open Access** This Peer Review File is licensed under a Creative Commons Attribution 4.0 International License, which permits use, sharing, adaptation, distribution and reproduction in any medium or format, as long as you give appropriate credit to the original author(s) and the source, provide a link to the Creative Commons license, and indicate if changes were made.
In cases where reviewers are anonymous, credit should be given to 'Anonymous Referee' and the source.
The images or other third party material in this Peer Review File are included in the article's Creative Commons license, unless indicated otherwise in a credit line to the material. If material is not included in the article's Creative Commons license and your intended use is not permitted by statutory regulation or exceeds the permitted use, you will need to obtain permission directly from the copyright holder.
To view a copy of this license, visit https://creativecommons.org/licenses/by/4.0/

**Response to referees**

I thank the reviewer for their comments on my Comment, "Study planning is better served by Monte Carlo simulation than AI-generated participants". Below, I reproduce each of the reviewer's comments, followed by my response.

**Reviewer 1**

> *The authors present a compelling argument for the transparency of MC simulations compared with the often opaque assumptions embedded in AI-generated "silicon samples." However, a central limitation of the current manuscript is its narrow definition of how Large Language Models (LLMs) function and are utilized by researchers.*
> *I strongly recommend addressing the following points to nuance the paper's core argument:*
> *The manuscript currently evaluates LLMs as if they were strictly in-context language generators, in which the model is prompted to directly output a synthetic dataset as text, relying solely on its internal weights and probabilistic next-token prediction. In this specific "naive" use case, the authors are entirely correct that the assumptions are buried, and the data-generation process is opaque. So the model did not generate or process any data, but just provided the most plausible anwer.*

**Response:** I thank the reviewer for their positive words. The reviewer is correct that the manuscript addresses the case in which the model is prompted to output responses directly. This is the specific use case of "silicon sampling" in the literature (in which model outputs are treated as the responses of simulated participants), which is the use case that the Comment is focused on responding to (e.g., Argyle et al., 2023; Ashokkumar et al., 2026). However, I agree that the manuscript did not previously make clear that other uses of LLMs fall outside this critique, and the new section now addresses this directly.

> *Or, did the model translate the prompt into python code and actually run a simulation in back-end? So, the output is based on an actual simulation study, only the parameters are not shown to the user. A simple prompt instruction will solve this problem:*
> *I would suggest that the authors acknowledge that modern LLMs are frequently used to generate, execute, and document actual code. If a researcher prompts a current-generation model to create a synthetic demographic dataset, a standard workflow is for the model to translate this prompt into a Python script, run a Monte Carlo simulation in the backend, and output the results alongside a fully documented GitHub repository, complete with scripts, adjustable parameters, and a README. All of this is stored at your laptop and be pushed to GitHub.*

**Response:** The newly added section now addresses the use of LLMs for coding assistance explicitly. However, the workflow the reviewer describes still leaves open the problem that the reviewer notes in passing, namely that the parameters are not shown to the user. In a simulation written by an LLM, the effect sizes, variances, and other parameters must still come

from somewhere; if they are selected by the model, they are of unknown practical relevance for the study being planned, in much the same way as a silicon sample estimate. The new section therefore also addresses this fact.

> *Because an LLM can trivially generate a transparent, reproducible Monte Carlo pipeline, treating "Silicon Samples via LLM" and "Monte Carlo Simulation" as mutually exclusive categories is a false dichotomy. The issue is not that LLMs cannot provide transparency; the issue is how researchers choose to interact with them. Therefore, my recommendation is to that the authors should consider shifting the focus of their critique toward researcher methodology. The paper would be significantly stronger if it cautioned against the "naive" use of LLMs for direct, unstructured data generation, while acknowledging that LLMs are highly effective tools for generating the very transparent Monte Carlo simulation scripts the authors rightfully advocate for.*

**Response:** The new section of the manuscript now addresses this point. I would note that the manuscript's critique was already directed at a methodological practice (i.e., treating LLM outputs as proxies for human quantitative data during study planning) rather than at LLMs as a technology, and the Conclusion lists several uses of LLMs in piloting which the critique does not touch. I hope that the new section makes this scope clearer for readers.

> *To strengthen the narrative, the authors might consider drawing a parallel between the current naive use of LLMs and the historical (and ongoing) misuse of p-values or Bayesian statistics. Just as a p-value is mathematically sound but frequently misused as a "black box" metric for truth (e.g., p-hacking) due to a lack of statistical literacy, LLMs are currently being misused as magic data generators. Similarly, Bayesian statistics require highly transparent justifications for priors. When researchers fail to document their priors, the Bayesian method becomes exactly what the authors fear LLMs are: an opaque container for unexamined assumptions.*

**Response:** I thank the reviewer for this suggestion. I found the Bayesian analogy well-fitting, and the new section now uses it directly.

> *Ultimately, I am afraid the core problem lies not with the tool, but, ingain with the human usr. Asen the ca,se with new sta,tistical tools, the danger lies not within the technology itself, but rather in how we as researchers choose to apply it.*

**Response:** I agree in part. Researcher practice is certainly a large part of the problem, and the new section places the responsibility for inspecting and justifying simulation assumptions with the researcher. However, I do not think that the problems of silicon sampling can be fully reduced to researcher literacy. Even a statistically literate researcher who uses a silicon sample for planning cannot inspect the data-generating process that produced it, nor determine why two equally defensible configurations give different estimates (see the section "Configuration dependence and generalizability in silicon samples"). This part of the problem is therefore a

property of the method itself rather than of how it is applied, and I have retained this argument in the revised manuscript.
